# Acute Muscle Trauma due to Overexercise in an Otherwise Healthy Patient with Cystic Fibrosis

**DOI:** 10.1155/2012/527989

**Published:** 2012-01-23

**Authors:** Henning Neubauer, Clemens Wirth, Katharina Ruf, Helge Hebestreit, Meinrad Beer

**Affiliations:** ^1^Department of Paediatric Radiology, Institute of Radiology, University Hospital Wuerzburg, Josef-Schneider-Straße 2, 97080 Wuerzburg, Germany; ^2^Department of Paediatrics, University Hospital Wuerzburg, Josef-Schneider-Straße 2, 97080 Wuerzburg, Germany

## Abstract

Cystic fibrosis (CF) is one of the most common inherited diseases and is caused by mutations in the CFTR gene. Although the pulmonary and gastrointestinal manifestations of the disease remain in the focus of treatment, recent studies have shown expression of the CFTR gene product in skeletal muscle cells and observed altered intramuscular Ca^2+^ release dynamics in CFTR-deficient animal models. Physical exercise is beneficial for maintaining fitness and well-being in CF patients and constitutes one aspect of modern multimodal treatment, which has considerably increased life span and reduced morbidity. We report on a case of acute muscle trauma resulting from excessive dumbbell exercise in a young adult with cystic fibrosis and describe clinical, laboratory and imaging characteristics of acute exercise-induced muscle injury.

## 1. Introduction

Life expectancy of patients with cystic fibrosis (CF) continues to improve. As randomized controlled trails demonstrate beneficial effects of exercise programs in CF patient, therapy increasingly focuses on building up and maintaining physical fitness and health [1]. Our report presents the case of a young adult with CF who suffered acute muscular injury as a result of overexercise, which demonstrates the necessity of moderate therapeutic muscle exercise at individually adapted exercise levels.

## 2. Case Presentation

Our patient, a 22-year-old male with cystic fibrosis, was admitted as an inpatient for scheduled intravenous antibiotic therapy of chronic pseudomonas airway infection. He was in good general health with 66 kg body weight, 184 cm height (BMI 19.5 kg/m^2^), and a constant Chrispin-Norman score (CN score = 7). Patient history included well-managed lung and gastrointestinal manifestations of CF, exocrine pancreatic insufficiency, impaired glucose tolerance, allergic rhinopathy, and nasal polyps. Occasional spare time sports activities were reported, although recurring air-way infections frequently prevented exercise. The treatment plan during the hospital stay included physiotherapy and supervised physical exercise.

### 2.1. Clinical Symptoms and Laboratory Findings

On day 6 in hospital, the patient suddenly complained about bilateral pain and swelling of upper arm muscles. Laboratory analysis revealed a marked elevation of previously normal CK, LDH, and CK-MB levels (Table 1). Serum creatine (<0.9 mg/dL) and glomerular filtration rate (150 to 181 mL/min/1.7m^2^) remained normal; C-reactive protein was negative (<0.10 mg/dL). The slightly elevated leukocyte count (<12.060/*μ*L) was clinically attributed to the presence of chronic airway infection and sinusitis.

### 2.2. Imaging Studies

**Figure 1:**
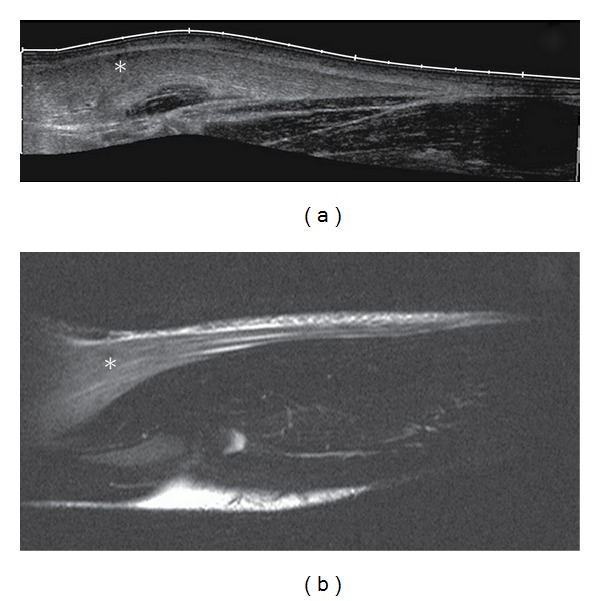
Ultrasound compound image (a) and corresponding sagittal MRI T2w TSE (b) of the right-hand elbow and forearm showing markedly increased echogenicity and corresponding signal increase of the brachioradial muscle (marked as *).

**Figure 2:**
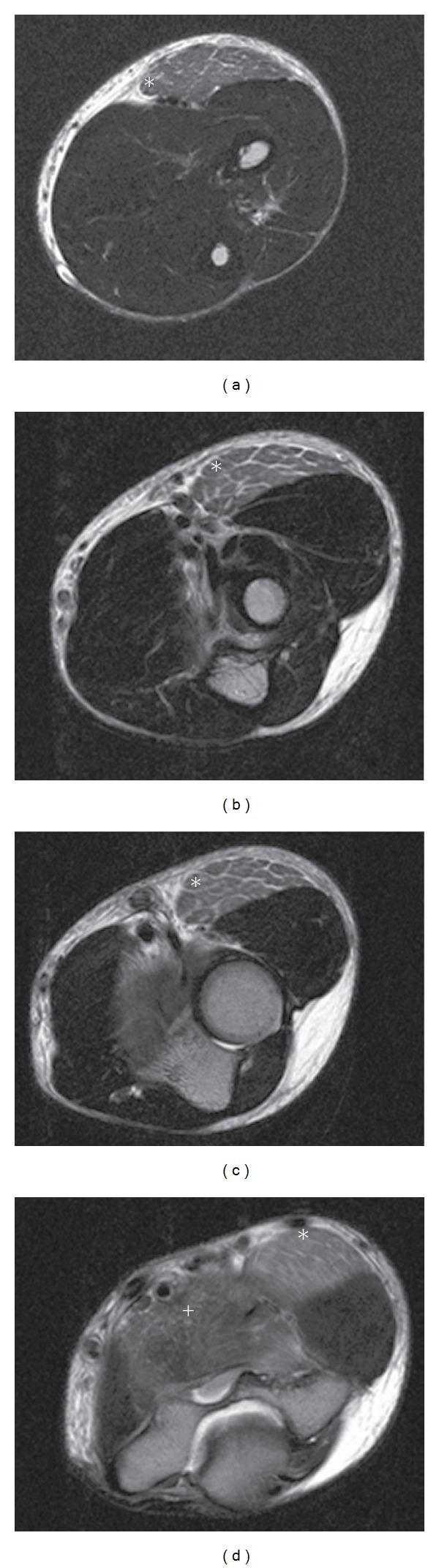
MRI of the right elbow (transversal T2w TSE) shows homogenous signal increase in the brachioradial (marked as *) and the brachial muscle (marked as ^+^).

As clinical symptoms persisted, an ultrasound examination was performed which revealed a diffuse bilateral increase in echogenicity limited to the biceps brachii, brachialis, and brachioradialis muscles (Figure 1). Normal echogenicity was seen in the pronator teres, flexor carpi radialis, triceps brachii, and deltoid muscles. Doppler ultrasound showed no signs of hyperperfusion in the hyperechogenic tissue. Deep vein thrombosis and thrombophlebitis were ruled out sonographically. MRI of the right elbow joint performed the next day confirmed the ultrasonographic diagnosis of edema confined to the described muscles with marked homogeneous signal increase on T2 TSE in the absence of other pathological signal alterations (Figure 2). The affected muscles showed normal signal intensity on T1-weighted images. Whole-body MRI scanning with coronal T2 TIRM and T1 TSE sequences did not reveal any other abnormal findings apart from a previously diagnosed pansinusitis (not shown).

### 2.3. Outcome

Upon interrogation, the patient admitted nonsupervised dumbbell training for more than four hours in his room one day prior to the onset of clinical symptoms, particularly exercising his “biceps” bilaterally. With immobilization and rest, clinical symptoms and laboratory findings normalized within days, so that no further imaging studies were performed. After completion of antibiotic infusion therapy, the patient was discharged in good health. Normal LDH and CK serum levels were seen on followup 4 months later (Table 1), and, with moderate levels of physical exercise, no recurrence of symptoms occurred.

## 3. Discussion

Physical workout beyond the lactate threshold induces transient physiologic muscle edema during, and briefly following, exercise in healthy individuals [2]. Overexercise may cause delayed-onset muscle soreness, a common symptom in recreational athletes, usually occurring within 24 hours after the overuse episode. Muscle sore may persist for days and even progress to rhabdomyolysis [3]. Muscle damage due to intense exercise leads to an increased muscle volume, a decrease in muscle force, and high CK values [4, 5]. Ultrasound examination shows an increase in muscle echo intensity and volume peaking at 4-5 days after exercise [5]. The extent of muscle edema, which is a focal or diffuse increase in intracellular and/or extracellular free water, can be visualized on fat-suppressed T2-weighted and inversion-recovery MRI sequences [6] with maximum signal alterations occurring about 7 days after exercise [4].

In our case, excessive exertion triggered the clinical onset of muscle soreness within 24 hours. Ultrasound and MRI, performed on the second and third day of clinical manifestation, both showed signal alterations suggestive of acute muscular edema. Differential diagnosis of diffuse muscular signal increase on T2-weighted images includes autoimmune conditions, such as polymyositis or dermatomyositis, infectious myositis, radiation therapy, subacute denervation and compartment syndrome, among others [7]. These conditions could be ruled out on the basis of patient history, clinical symptoms, laboratory findings, and spontaneous recovery. The distribution of the homogenous signal increase on T2w, bilaterally confined to muscles performing elbow flexion and forearm supination, matched the reported mode of dumbbell exercise in our patient. Abnormal pattern of signal intensity indicative of muscle disruption or intramuscular hematoma were not observed. The diffuse signal increase of the affected muscles together with the marked elevation of CK, CK-MB and LDH serum levels allows the diagnosis of acute exercise-induced muscle injury.

Recently, Divangahi et al. located CFTR protein in human and murine skeletal muscle cells and demonstrated altered contractile function with force loss, dysregulated calcium homeostasis, and proinflammatory response in diaphragm cells of CFTR-deficient mice with pseudomonas lung infection [8]. Furthermore, there are data indicating abnormal pattern of high-energy phosphate turnover, as measured with 31P-magnetic resonance spectroscopy [9]. So far, there are no published reports on a particular proneness to exercise-induced muscle edema in CF patients, leaving the question open whether the manifestation of postexertional muscle damage may or may not be more severe in these patients than in healthy individuals. The etiology of postexercise muscle soreness is generally not well understood and is usually attributed to muscular acidosis and microtrauma. In healthy individuals, this condition is commonly seen in response to strenuous exertion exceeding accustomed exercise levels or deviation from accustomed pattern of exercise. One may hypothesize that CF patients, generally less physically active in daily life, are more susceptible to muscular edema secondary to exercise-induced microtrauma and altered muscular microenvironment.

A review of literature provides evidence that CF children benefit from exercise programs in terms of improved fitness, strength, and pulmonary function [1]. Morphological and functional MR imaging studies systematically investigating postexercise muscular signal changes in CF patients, compared with healthy peers, may help to define adequate exercise levels and avoid muscle damage.

## Figures and Tables

**Table 1:** Laboratory findings. Course of LDH (reference range < 250 U/l), CK (reference range < 190 U/l), and CK-MB serum levels. The traumatic exercise occurred on March 30th. Ultrasonography was performed on April 2nd, MRI on April 3rd.

	LDH	CK	CK-MB
	U/l	U/l	U/l
09.05.2005	192		
13.05.2005	170		
30.09.2005	189		
28.01.2007	155		
23.08.2007	148	84	
29.09.2008	231		
27.03.2009	191		
31.03.2009	1954	44600	327,9
01.04.2009	1874	58372	378,6
02.04.2009 (US)	1302	41017	
03.04.2009 (MRI)	484	18507	
04.04.2009	314	7296	
05.04.2009	185	2783	
07.04.2009		322	26,4
08.04.2009	179	461	
17.04.2009		162	
20.08.2009	234	149	
